# A socially prescribed creative play intervention for new parents: investigating post traumatic stress around birth and changes in postnatal depression and reflective function

**DOI:** 10.1186/s40359-025-02578-3

**Published:** 2025-03-23

**Authors:** Paige E. Davis, Susanna Kola-Palmer

**Affiliations:** 1 Department of Psychology, Leeds University, Woodhouse, Leeds, LS2 9JT UK; 2 Department of Social and Psychological Sciences, University of Huddersfield, Queensgate, HD1 3DH UK

**Keywords:** Social prescribing, Depression symptomology, Reflective functioning, Parenthood, Creative play, Birth trauma

## Abstract

**Background:**

Parenthood is a key transition period which involve emotional, social and physical adjustments. Social prescribing is a method that connects people to community-based activities, groups, and services to addressing various needs impacting their health and wellbeing. This pilot investigation aimed to assess whether a curated socially prescribed creative play programme would impact upon new parents’ social connection, mental health and reflective function through a programme designed to support these changes.

**Methods:**

This study was part of a 5-week long socially prescribed creative play programme at a family theatre company in the North of England, aimed at providing social capital to families while teaching creative play. In total, 57 parents (*M =* 30.73, *SD* = 6.20) completed baseline and post-intervention measures of birth trauma experiences (City Birth Trauma Scale), postnatal depression (Edinburgh Postnatal Depression Scale) reflective function (Reflective Functioning Questionnaire), and qualitative, open-ended questions on social opportunities. Descriptive analyses were completed using t-tests and chi-square tests, while repeated measures ANOVAs were used to answer questions around the main analyses.

**Results:**

The participants experienced a statistically significant reduction in postnatal depression scores following the intervention, but no changes were found in reflective function or birth trauma scores; secondly, birth trauma scores predicted later depression scores as well as reflective functioning uncertainty scores (but not certainty scores). Qualitative analysis found social opportunities were not why parents came but was, after attending, their favourite part of the socially prescribed programme. Those parents reporting on social opportunities were more likely to reference their own needs while non-social activities were associated with their child’s needs.

**Conclusions:**

Socially prescribed creative play programmes for new parents could be a “waiting well” intervention. A longer duration and trauma informed focus would need to be considered in future cohorts.

**Supplementary Information:**

The online version contains supplementary material available at 10.1186/s40359-025-02578-3.

## Background

### Helping parents through critical life transitions

New parenthood is a key transitional time in the lifespan filled with changes in identity, social support, physical and mental health and these shifts can bring stress and impact wellbeing which has direct implications for children [1–5]. New parents are susceptible to postnatal depression and can become more vulnerable if they have had a traumatic birth, experience less social support or find it hard to understand their own or others mental states [6–8].

### Birth trauma and postnatal depression

One area that parents in particular are affected by is birth trauma experiences and the post-traumatic stress disorder (PTSD) which may result [9]. Psychological birth trauma has been found to be on the rise and with its implications for mental health and parenting, reporting of birth trauma could be one potential risk factor for vulnerable parents [10]. Birth trauma-related PTSD can evolve after having negative emotions, loss of control and distress during labor and delivery [11]. Other risk factors include fearing for one’s own life or the life of the baby [11]. PTSD originating from traumatic birth experiences is related to poor coping and stress and up to 25% of mothers can experience clinical levels of PTSD regardless of whether they have had a healthy baby at term or not [12, 13]. PTSD symptoms expressed in new mothers are often times comorbid with depression symptomology [14]. Together these mental health issues can impact attachment behaviours, the mother’s overall wellbeing and her child’s health [14].

New parenthood is also a period that is also widely recognised as a time of high risk for postnatal depression regardless of whether birth trauma was experienced [15, 16]. Depending on the context and culture, postnatal depression has been reported to be experienced by 6.5–12.9% of new parents globally [17, 18]. Postnatal depression not only impacts parents at the time they are experiencing it but has also been found to be associated with long term effects on child development like poor school performance compared to peers [19], and social and behavioural issues at school age [20]. Postnatal depression has also been found to relate to child internalizing problems at age six [21].

### Parental well-being and social support

Becoming a parent often reduces the size of social networks, with new parents reporting having fewer friends and less social support [22]. Social support has been found to have a strong relationship with the stress that a parent feels. Researchers suggest that it appears to have an essential role in parental well-being and family adaptation [23]. Social isolation and lack of social support may lead to poorer outcomes for mothers and children [24, 25]. Social support is a protective factor against parenting stress and coping difficulties during the transition to parenthood [26].

There are a variety of interventions for new parents targeting different areas of potential stress or building on well-known protective factors (e.g. 4, 9). Intervention can range from programmes like online psychoeducation around parenthood, to in person peer support for infant feeding [27, 28]. Intervention for postnatal depression in specific also comes in many forms and a host of interventions consider variables like social support and birth trauma experiences and how parents think about their infants (e.g. 9, 12, 13). One social support intervention that has been found to be helpful to parents links them with nurse partners who ask if they have concerns about their babies health and follow up numerous times [29]. Other social support interventions include individual therapies, home visiting and internet support [30].

There is no evidence in the literature that any group interventions for new parents focus on all of these variables while also engaging the infants simultaneously. Furthermore, there are not any socially prescribed (SP) interventions for new parents focusing on creative play for new parents in the United Kingdom. This study was designed to determine if an SP creative play programme for new parents and their infants focusing on these four key variables would impact parental postnatal depression.

### Social prescribing

SP is a strategy to mediate the increasing mental health and wellbeing needs of individuals by providing members of the public with psychosocial issues access to community non-medical support instead of putting pressure on healthcare systems [31, 32]. SP can vary widely in terms of the activities provided (from arts on prescription to archaeology on prescription), but its overarching goal is to support the most vulnerable individuals through interventions adapted to fit target groups [33, 34]. The interventions are person centred and aim to simultaneously reduce isolation and improve psychological wellbeing [34]. Outcomes of SP range from improved mental health, wellbeing and depression, to lessening social isolation through peer-to-peer support [35–37].

SP has been found to facilitate life transitions, and past research has indicated new parenthood to be a critical time where health and social interventions could impact changing health behaviour [38]. Furthermore, new parents are at a stage where they are susceptible to specific elements contributing to health disparities that SP creative play programmes could address [38, 39]. Reports of mental health issues within the perinatal period have significantly increased since the Covid-19 pandemic [15] and there is a need to understand how to most effectively reconcile these issues.

New families with children 0–3 years are a demographic rarely thought of as a target for SP even though transition to parenthood and infancy relate to key components of SP like social support from isolation and a need for health and wellbeing signposting. A SP creative play programme could be one way to implement community support that facilitates positive changes in the key areas of social support, mental health and wellbeing, but also would be able to impact both parent and infant.

### Creative play

Play sessions are often seen in the community, so developing this type of intervention seems well placed for families in this timeframe as this is also a time where infants are building attachment relationships and learning about the dynamics of social environments [40]. Play has been found to relate to and foster caregiver-child attachment and other development such as social, cognitive and emotional skills [41–43]. During play, infants are learning relationships with varying communication behaviours while infant and adult brains are linked through social cues such as speech prosody or joint attention [40, 44]. There is a recognition that SP in general will promote positive health outcomes for infants and very young children. A major commissioner and a large children’s charity in the United Kingdom have both called for SP programmes for youngsters to be established in every area of the country [45, 46].

While playgroups are a well-known form of intervention, there are advantages to a SP creative play programme above and beyond a typical playgroup model [47, 48]. Having a professional artist practitioner that is trained in both the arts and child development combined with a creative play focus has been found to instill in parents a sense of calm and a feeling of non-judgement [47]. There is also a host connecting participants, introducing the creative play activities and making them a drink. Throughout each session, parents get information on how the play activity is helping their infant to develop. This atmosphere encourages health and wellbeing signposting and peer support networks within the group [47]. Past research on play interventions has found that these interactions can have a significant role in reducing parental anxiety [49], however more research is needed around carer and family outcomes as most research on play is based on children [50].

Other interventions with no creative play element that have been found to have positive mental health impact on carers include first-time parent groups which have reported enduring benefits of increasing self and social confidence as well as access to information on infant health [51]. Periodic newsletters have also been a cost-effective way to prepare carers with parenting skills and improve the ability of new parents to play with their infant, however this study had implications for reaching vulnerable populations because of the emphasis on reading [52]. Bearing this in mind, the first SP creative play intervention was run and evaluated in 2021. This programme was changed and refined through the evaluation to focus on enriching the parental experience.

### Intervention around birth trauma and vulnerable parents

Because of the format of SP, the programme itself was not specifically oriented for carers experiencing birth trauma, however referred parents with PTSD resulting from birth may be targeted by prescribers because of the comorbidity with depression [53]. The creative play intervention did not aim to “fix” these issues, but rather it was a programme that was curated to be a safe place to talk about these feelings and behaviours with other parents (see 18). There was acknowledgement that these parents may be amongst the most vulnerable in the group. The hope was that through peer support, parents would begin to process their trauma if they indeed had experienced a difficult birth.

### Parental wellbeing and play intervention

SP has been found to benefit populations who are lonely or socially isolated, and those at high risk of mental health problems, which are all issues new parents face [34]. Furthermore, the type of intervention chosen was deemed to be a good fit for the population because of the normalisation of “playgroups” [48]. Some researchers would argue that SP doesn’t have a clear evidence-based theory to explain its efficacy, however the ‘social cure’ fits well with this intervention [54]. The social cure theory explains how group membership can be a source of social support under conditions where individuals are stressed or ill influencing a person’s well-being and health [55]. Even general practitioners have been found to recognise the limits of the medical model and the place for SP to address social needs in the community [56].

There are three related areas of parental health and wellbeing that the SP creative play intervention aimed to impact (social support, postnatal depression and reflective function (RF)). These areas have been found to intertwine and the hope for the SP programme on a whole was that changes in some of these variables might impact upon others [7, 57–62]. Play is an inherently social activity and engagement in playful activities has been found to impact parental depression [21, 43, 63, 64]. Play also has been theorized to be a precursor to later mentalisation and RF [65].

### Intervention to improve social support networks

First, opportunities for social capital and social support were encouraged through the specialist artist practitioner and the host as these had been identified as areas that were important to parents and their mental health [24, 47, 58]. This was also backed up by the evidence that peer support and relationships can provide long-lasting benefits to new parents and their families [51]. Furthermore, social networking uniquely contributes to positive health outcomes making it a critical behaviour in the postpartum period [59, 66]. Parents, especially mothers, are an at-risk group for social isolation which is linked to the exacerbation of cardiovascular disease and depression [24]. Social support in both new mothers and fathers relates to postpartum depressive symptomology [8, 67]. Connecting parents through an SP creative play programme could increase social support and connection, especially after the COVID-19 pandemic has impacted new parents’ mental health, socialization and wellbeing [15]. Parents’ concerns while in isolation were socialization for themselves as well as their children, and some parents reported online support could not replace in-person offers of informal and formal support [68].

### The intervention’s focus on postnatal depression

The second area of focus was postnatal depression. SP creative play was created to focus on parental mental health and depression by building parenting confidence, alleviating stress and normalizing feelings for parents [66]. Each session was divided into 3 unequal segments and one of those segments involved a semi-structured creative play offer where parents could join in with other parents and discuss anything that was on their mind (a table of topics discussed in these sessions can be found in [47]). Throughout the sessions the host and artist practitioner would come around and give words of encouragement. The carers would be signposted to behaviours that would improve bonding with their infant as postnatal depression has been shown to be interconnected with reduced mother infant bonding [60].

However, research has found that parent bonding is able to mediate the relationship between postnatal depression symptoms and child behavioural problems [60]. Furthermore, according to some findings, direct intervention has been suggested to be able to facilitate improved mother-infant relationships in only four mother infant sessions [69–71]. If these follow-on child issues could be prevented through learning about and providing space to bond, it could save money for the national health service in the long term.

### Reflective function’s place in the intervention

The final area that has been focused on through this intervention is RF, which is the skill of understanding one’s own and others’ mental states (e.g. 47). Specifically, it relates to the ability to understand and interpret one’s own and others’ behaviour as reflecting mental states, such as thoughts, feelings, and beliefs [72, 73]. Research using a general measure of RF has found evidence of poorer family functioning and increased anxiety and depression symptomology in caregivers of children [74], and RF has been found to be impacted by trauma [62]. RF, therefore, may allow parents to become attuned to infant’s needs and mental states in order to understand and engage with them effectively [57]. This enables parents to be able to meaningfully relate to their own children’s behaviours through this consideration of their children’s inner states [75]. It also allows parents to engage in less emotionally charged interactions with their children when they are dysregulated [57], and later on these children have been found to have less internalizing and externalizing issues [76–78]. Parents who are unable to use RF have been found to have lower quality relationships with their children [79]. Moderate improvements in RF have been found when interventions stressing mentalization and dyadic interaction are run with high-risk parents [6]. Parental RF can promote their child’s own reflective thinking in the future and parents with higher levels of RF have been found to support their children’s playfulness [80]. For this reason, the SP intervention focused on infant/child-parent/carer interactions and stressed thought about what the dyads were thinking while singing and playing.

### Aims and hypotheses

There is literature exploring birth trauma, social support, postnatal depression and RF, however no literature currently examines whether the mental health of carers with 0–3 year olds is impacted specifically by a child-centred SP creative play intervention. Thus, this pilot investigation aims to look at the efficacy of the creative play on prescription programme in terms of parental health and wellbeing. Specifically, it will examine:

1. Whether there are social opportunities experienced by parents attending the creative play programme.
2. If there is a relationship between parental report of birth trauma and depression and reflective function.
3. If the report of postnatal depression symptomology decreases from baseline to after the 5-week creative play intervention.
4. If the report of parental reflective function improves from baseline to after the 5-week creative play intervention.

Based on previous literature it was hypothesised that higher scores of birth trauma would be predictive of higher depression symptomology, and lower scores of RF. Because of the investigatory nature of the research we hoped that depression symptomology would decrease and RF scores would improve from baseline to after the SP intervention. There were no directional hypotheses made around the social opportunities as this was an aim that would be assessed qualitatively.

## Methods

### Study design and setting

This study was a part of a 5 week SP play programme aimed at providing social capital to families while teaching play. Each session was divided into three parts; guided singing, curated creative play opportunities, and a transitional winding down song before leaving. Session descriptions can be found in [47, 81]. Stakeholders’ perceptions of the programme can be found in [82].The programme was carried out in a family theatre company based in the North of England. In order to support the aims of the study a quantitative and statistical approach was employed using a baseline (T1) and post intervention survey (T2). Scores from three main measures were utilized at both timepoints.

### Participants

Parents came from 3 separate cohorts enrolled in the same 5-week creative play on prescription programme. There were 57 parents aged between the ages of 17 and 42 years (*M = 30.73*,* SD =* 6.20) that took part in either T1 and T2 surveys. There were 27 parents taking both (*M = 30.59*,* SD =* 5.50). Most parents, *n* = 41 (84%), identified as white British or white, with *n* = 8 identifying as belonging to a minority ethnic group. Incomes ranged from estimates of £10,000 per year to over £89,000.

This study was approved by the university ethics committee. Approval code: ETH2223-0080. Participants were recruited from each cohort beginning March 2nd, 2023, and concluding 1st of December 2023. Recruitment for the programme was facilitated by the organisation running the SP intervention. In line with the NHS guidelines, parents arrived through two referral pathways; health worker/stakeholder, or self-referral (NHS Social Prescribing, 2021). To meet criteria for self-referral, parents and carers needed to tick a referral questionnaire explaining that they had “low confidence”. Parents also needed to have a child that was between 0-3-years to be invited to the programme. Only parents enrolled in the SP groups met the inclusion criteria for this study.

In the first session, all members were informed of the study and given the opportunity to either complete the first questionnaire at the organisation or go home and fill it out. This enabled researchers to ensure parents who may not want to take the survey would not feel pressure. All participants were given information sheets and provided written consent before commencing each questionnaire. In addition to the informed consent, the parents participating also were given a debrief with resources in case of potential distress. Parents had the option of providing a unique code with which they could remove their data if they chose.

### Materials and social prescribing intervention

#### The intervention

The SP creative play intervention took place every Thursday for 5 weeks. It was designed around the overarching ethos of “follow the fascination” which explores creativity both as a creative experience and a product, following the infant or child’s lead. Music was viewed as a type of play where parents/carers and infants could engage together. For more information around the ethos and creative theory that the programme was based on, please see [81, 83].

It consisted of three allocated time slots throughout the day where parents were enrolled for sessions. Parents were grouped into these time slots according to their child’s age. During each session parents were greeted by a professional arts practitioner and a host. Parents with younger children attended the early time slot. This was less structured but did include guided imaginative singing and creative play. The two slots with older children were structured beginning with the same facilitated songs as a group and followed by a free play session where parents were signposted to speak with each other while playing with their child. Free play was followed by a 15-minute guided circle-time where families would sing and be given a pamphlet with activities for the week. They would learn about their children’s development while doing one of the activities suggested. Sessions would close with a goodbye song. For more in depth information on the sessions set up see [47, 81].

#### The surveys

Two surveys were distributed for each cohort. The baseline T1 survey was given in the first session and a shorter, post intervention T2 survey was distributed in the final session. Participants were informed that if they would like to participate, they could complete the survey on paper, or through a QR code or link. If the survey was completed electronically it could be with a provided tablet or their own device. The T1 survey consisted of 5 parts following the information sheet and consent questions. These were, (1) demographics and parenting practice, (2) reflective function, (3) postnatal depression symptomology, (4) birth trauma, and (5) a referral and qualitative question. The T2 survey also contained 5 sections but was shorter than T1 leaving out some of the birth trauma questions that were previously answered. Sections for each survey are detailed below.

### Demographics and parenting practice

The demographic section asked questions about the gender identity and ages of the parent and their children. Their estimated family income, ethnicity and whether they had come to a previous iteration of the intervention.

### The reflective functioning questionnaire [84]

The RF questionnaire was developed to measure mentalizing ability [84]. It is an 8-question self-report measure using a 7-point Likert scale from 1, strongly disagree to 7, strongly agree. There are subscales for certainty and uncertainty about self and others’ mental states. An example of a certainty question would be, *“people’s thoughts are a mystery to me”* where an uncertainty question would be, “*strong feelings often cloud my thinking*.” Higher RF was indicated with higher scores. T1 alpha scores for certainty were acceptable at 0.75 while T2 alpha was good at 0.89 uncertainty at T1 was low at 0.62 and acceptable at T2 0.78, and total reliability analysis at T1 indicated a low alpha score at 0.68 and a good score of 0.85 at T2.

### The Edinburgh postnatal depression scale [85] (EPDS)

The EPDS is a validated questionnaire used widely in the field to assess postnatal depressive symptomology in parents and their partners [58, 85, 86]. The questions are based on how the participant felt in the past 7 days. This measure has questions relating to different depression symptomology. Questions like, *“I have felt scared and panicky for no very good reason”* cover symptoms related to worry and panic, while the questions like, *“Things have been getting on top of me”* relate to coping and *“I have felt sad or miserable”* relate to low mood. The questions are all measured on a 4-point Likert scale. Scores ranged from 0 indicating no symptomology to 3 indicating the highest level of symptomology with a final range of 0–30. The reliability for the EPDS was excellent, 0.89 at T1 and 0.92 at T2.

### The City birth trauma scale [9]

This is a validated measure which is intended to capture birth-related post traumatic stress disorder (PTSD) symptomology [9]. There are 31-items in the scale, 29 of which map onto the DSM-5 diagnostic criteria for PTSD. The first three questions ask when the birth occurred and go on to ask about whether the parent believed that they or their baby would be seriously injured or die during the labour, birth or immediately afterwards. These questions are followed by 10 symptom related questions about the birth where respondents are asked to indicate their experiences of symptoms in the last week on a response scale ranging from 0 indicating *not at all* to 3 indicating *5 or more times*. The questions ask about flashbacks (e.g. *Flashbacks to the birth and/or reliving the experience*), intrusive thoughts (e.g. *Recurrent unwanted memories of the birth (or parts of the birth) that you can’t control*), and nightmares (e.g. *Bad dreams or nightmares about the birth (or related to the birth)*) amongst other symptomology.

After that, 12 questions about symptoms that began or got worse since the birth. These questions area also linked to the DSM-5 diagnostics and relate to symptoms like concentration (e.g. *problems concentrating*) and feeling detached (e.g. *Feeling detached from other people*). Two follow up questions target when symptoms began (whether they began before the birth during the first 6 months, or more than 6 months after the birth) and how long they have lasted (less than 1 month, 1 to 3 months, or 3 months or more). The final group of three questions focuses on impact of the symptoms causing distress, preventing daily activities and whether the symptoms could be due to other medical or non-medical reasons. The reliability for this scale at T1 was excellent at 0.95.

### Let’s play thoughts

The final four questions were qualitative in nature, asking about how the participants were referred to the programme and what interested them most about the programme. There was a question on whether they thought the SP programme was different than a typical play group, and a chance to explain whether they thought it was particularly social, judgmental, good for mental health, or stress.

### Statistical methods

All analyses were completed using *Statistical Package for the Social Sciences* version 29. After data imputation possible covariates were analysed. After preliminary descriptive analysis, t-tests and chi-square test of independence were used to investigate whether there were group differences in those who dropped out of the intervention. A repeated measures ANOVA was employed to determine whether there were significant changes in postnatal depression and RF between T1 and T2. Whether the parent had been to a previous cohort was considered as a between-subjects factor. Linear regression analyses were used to determine whether birth trauma impacted depression or RF scores after the intervention. Correlations were run between the main variables and covariates to look at associations between the variables.

### Content analysis

The qualitative questions were examined through a content analysis. The researchers looked at questions pertaining to what initially interested parents in the SP programme and what their favourite part was about the programme. These answers lent themselves to social and non-social narratives. Thus, the questions were coded for social themes and then separately coded as directed at the child’s needs, the parent’s needs, or both.

The second pair of questions used in the content analysis were those about what made it different from typical ‘play groups’ and how SP creative play helped the parents. The first of these questions was used in Davis and colleagues [47] and both were coded to reflect the three themes of trust, calm and practical parenting knowledge which the paper found. The themes of music and one of the groups being intimate, child-led and personalised were also coded, as this was also considered to be important components of the intervention and upon looking at the corpus of data, these were prevalent in the content of the responses. For examples of these codes see Table 1.

**Table 1:** Examples of responses for the content analysis

Pair #	Analysed theme	Question	Quotation example
1	Social Narrative	First Interest	*The idea of getting out of the house with little one to interactive and socialse with other people*
		Favourite Part	*Enjoyed meeting new people*,* small group + getting to know them. Fun activities for children*
	Parent Needs	First Interest	*Meeting others*,* something fun to do*
		Favourite Part	*it gives me a small break + it is free*
	Child Needs	First Interest	*My child does not speak trying to bring him on.*
		Favourite Part	*Seeing my daughter build relationship with other people and something she really looks forward to every week.*
	Both Parent and Child Needs	First Interest	*This session includes singing together and interaction section. So I thought it might be useful for him (my son) who got delay in speech and language. Also we both can enjoy some good time together in a peaceful setting.*
		Favourite Part	*interacting and socialising with other parents my child socialising.*
2	Trust	Differences	*Yes*,* L is able to be himself as he is being assessed for autism at other groups sometimes I am uncomfortable*
		Help	*I am currently pregnant with my second baby so have found some really good connections and friendships for other people in the same position as me. been a relaxed environment with no judgement to share experiences and worries.*
	Calm	Differences	*relaxed*,* non-judgemental*,* supportive -my boy feels safe/at home*
		Help	*I have always struggles with anxiety in social situations*,* however events like this make me feel more comfortable.*
	Parenting Knowledge	Differences	*Gives you ideas of things you can do with baby at home so it doesn’t just stop with the session*
		Help	*Parents with children at similar stage so able to discuss e.g. toddler routine*,* the sessions helped show there’s no right or wrong just use every interaction with baby as bonding time and always encourage them and continued positivity*
	Creative Music and Play	Differences	*Nice and related. My child was engaged and enjoyed himself he remembered the new songs when we got home*
		Help	*Loved seeing my child engaging and playing with other grown ups and babies*,* felt supportive and non judgmental (although I probably sing louder at home!)*
	Intimate and Child Focused	Differences	*Yes much more relaxed and less overstimulating for little ones. More intimate.*
		Help	*Smaller group more chance to talk…I had coffee in fed -This group FAB for mental health. Need more mental health struggling mums to attend.*

## Results

### Preliminary analysis

Out of 57 participants, 27 completed T1 and T2, 23 completed T1 only, and for 7 it was not possible to match T2 data with baseline data. Working with the missing data, preliminary analysis showed that there are no systematic differences between those who completed both T1 and T2 assessments and those who completed T1 assessments only in terms of demographics (age, child age, income, and ethnicity; see Table 2), birth trauma or outcome variables (reflective function, and postnatal depression; see Table 3). Differences between those who attended the creative play sessions for the first time and those that had also attended previous intervention cohorts were investigated. There were no differences between groups on measures of birth trauma or outcome variables (reflective function, or postnatal depression), see appendix 1 Tables 1 and 2 for a summary of these results.

**Table 2:** Sample characteristics with chi-square analyses to examine completion differences

Variables here	Total		Both		T1 only		X2
	*n*	%	*n*	%	*n*	%	
Child gender							
Male	28	56	15	55.6	13	56.5	0.005
Female	22	44	12	44.4	10	43.5	
Income							
< 29k	17	39.5	11	40.7	6	37.5	0.04
> 30k	26	60.5	16	59.3	10	62.5	
Ethnicity							
Majority	41	83.7	22	84.6	19	82.6	.58a
Minority	8	16.3	4	15.4	4	17.4	
Previous intervention							
Yes	17	37	14	56	3	14	8.52**
No	29	63	11	44	18	86	

Note * *p* <.05, ** *p* <.01, *** *p* <.001

a= Fisher’s exact test

**Table 3:** Mean differences in those who completed both assessments and those who only completed time 1 assessments

	Time 1 and 2 (*n* = 27) Mean (SD)	Time 1 only (*n* = 21) Mean (SD)	t statistic (df)	*p* value
Parent age	30.59 (5.50)	30.90 (7.16)	-0.17	0.87
Child age (months)	12.00 (7.89)	15.00 (9.41)	-1.18	0.25
Sum EPD	10.81 (5.15)	10.57 (5.31)	0.17	0.88
City Bits PTSD Symptoms	8.78 (7.92)	8.00 (8.36)	0.32	0.75
Sum RFQ Certainty	0.97 (0.78)	1.15 (0.68)	-0.83	0.41
Sum RFQ Uncertainty	0.56 (0.58)	0.53 (0.57)	0.20	0.84

There were no correlations between parent age and RF, postnatal depression or birth trauma at T1 or 2. There were also no correlations between child age and the main outcome variables for the missing data. See this table in appendix 2.

### Postnatal depression and reflective functioning scores

The EPDS and the two types of RF (certainty, uncertainty) scores were analysed in a repeated measures ANOVA to determine whether the means differed significantly from T1 to T2. The intervention status (new, previously experienced) was added in as a between-subject factor to determine whether there were any interactions between these variables. There were statistically significant differences in EPDS scores, *F* [1, 23] = 16.39, *p* <.001, ηp^2^ = 0.416, indicating a significant reduction in depressive symptoms from T1 to T2. There were no significant between-subjects effects of intervention group status, *F* [1, 23] = 1.67, *p* =.209, ηp^2^ = 0.068 or interactions *F* [1, 23] = 0.07, *p* =.796, ηp^2^ = 0.003 on EPDS scores. There were no statistically significant differences in certainty scores for RF, *F* [1, 24] = 2.20, *p* =.151, ηp^2^ = 0.084, although means rose from T1 to T2. There were no statistically significant between-subjects effects *F* [1, 24] = 0.26, *p* =.614, ηp^2^ = 0.011 or interactions with intervention status *F* [1, 24] = 2.20, *p* =.151, ηp^2^ = 0.062 for RF certainty. No statistically significant differences were found in uncertainty scores between T1 and T2, *F* [1, 24] = 2.49, *p* =.128, ηp^2^ = 0.094. There were no statistically significant between-subject effects *F* [1, 24] = 0.124, *p* =.728, ηp^2^ = 0.005, or interactions *F* [1, 24] = 1.61, *p* =.217, ηp^2^ = 0.063 for RF uncertainty. The analysis can be found in Table 4.

**Table 4:** Results of Repeated-Measures ANOVA

Dependent variable	Effect	F	df	*p*	Partial η	Interpretation
EPDS	Main Effect of Time	16.39	1, 23	< 0.001	0.416	Significant decrease in depressive symptoms from T1 to T2.
	Main Effect of Intervention Status	1.67	1, 23	0.209	0.068	No significant difference between intervention groups.
	Interaction (Time × Status)	0.07	1, 23	0.796	0.003	No significant interaction effect.
RF Certainty	Main Effect of Time	2.20	1, 24	0.151	0.084	No significant change over time.
	Main Effect of Status	0.26	1, 24	0.614	0.011	No significant group effect.
	Interaction (Time × Status)	2.20	1, 24	0.151	0.062	No significant interaction effect.
RF Uncertainty	Main Effect of Time	2.49	1, 24	0.128	0.094	No significant change over time.
	Main Effect of Status	0.12	1, 24	0.728	0.005	No significant group effect.
	Interaction (Time × Status)	1.61	1, 24	0.217	0.063	No significant interaction effect.

**Table 5:** Linear regression analysis of postnatal depression scores at T2

	*R*	*R* ^2^	B	SE	β	t
	0.83	0.68				
T1 EPDS score			0.63	0.14	0.61***	4.42
City Bits Birth Trauma PTSD Score			0.11	0.05	0.32*	2.31

Note Statistical significance: * *p* <.05, ** *p* <.01, *** *p* <.001

### Birth trauma scores

A linear regression with the birth trauma PTSD score as the predictor variable and EPDS score after intervention as the outcome variable, controlling for T1 EPDS score showed PTSD score significantly predicted EPDS *F* [2, 24] = 25.73, *p* <.001, adjusted R2 = 0.66. A linear regression with the birth trauma PTSD score as the predictor variable and time 2 RF uncertainty scores as the outcome variable, controlling for T1 RF uncertainty scores showed PTSD score significantly predicted T2 RF uncertainty scores, F [2, 25] = 11.20, *p* =.001, adjusted R2 = 0.43. However, PTSD originating from birth trauma did not predict RF scores of certainty *F* [2, 25] = 10.28, *p* =.001 at T2, when controlling for T1 RF certainty scores. See Tables 4, 5 and 6.

**Table 6:** Linear regression analysis of reflective functioning - certainty scores at T2

	*R*	*R* ^2^	B	SE	β	t
	0.67	0.45				
T1 RFQ Certainty			0.61	0.18	0.57**	3.48
City Bits Birth Trauma PTSD Score			-0.01	0.01	-0.19	-1.21

Note Statistical significance: * *p* <.05, ** *p* <.01, *** *p* <.001

### Correlations between PTSD from birth trauma, postnatal depression and reflective function

Bivariate correlations were run to investigate the main variables further. All variable combinations related significantly to each other, except for RF certainty scores at T1 and the EPDS at T2 *r* = −.281, *p* =.155. For all correlations see Table 7.

**Table 7:** Linear regression analysis of reflective functioning - uncertainty scores at T2

	*R*	*R* ^2^	B	SE	β	t
	0.69	0.47				
T1 RFQ Uncertainty			0.31	0.18	0.31	1.73
City Bits Birth Trauma PTSD Score			0.02	0.01	0.46*	2.59

Note Statistical significance: * *p* <.05, ** *p* <.01, *** *p* <.001

### Content analysis

Participants were first interested in the SP programme for both social and non-social reasons, however when it came to their favourite part of the programme, more parents cited social reasons for liking the intervention. When it came to social orientation, parents seemed more prone to cite their own needs as the focus than their children’s and this flipped when non-social orientation was mentioned in the responses. This was confirmed through a chi-square test for independence examining the relationship between social orientation and needs. The association between these variables was statistically significant and moderate, *X*^2^ [2] = 10.53, *p* =.005, Cramer’s *V* = 0.40, with a particular emphasis on child needs when the orientation was not social. In terms of the themes in the second pair of questions the theme encompassing the group being intimate, personalised and child-led was the main group difference reported and parents found they trusted the group feeling that there was no judgement and felt calm throughout. The music and play were also main themes. Content analysis findings can be seen in Tables 8 and 9.

**Table 8:** Correlation table for birth trauma, postnatal depression and reflective function

Variable	*n*	1	2	3	4	5	6	7
1. Birth Trauma Scale T1	45	__						
2. EPDS T1	45	0.569**	__					
3. EPDS T2	27	0.650**	0.781**	__				
4. RFQ Certainty Scores T1	45	-0.420**	-0.467**	-0.281	__			
5. RFQ Certainty ScoresT2	28	-0.431*	-0.715**	-0.567**	0.648**	__		
6. RFQ Uncertainty Scores T1	45	0.478**	0.645**	0.486*	-0.680**	-0.576**	__	
7. RFQ Uncertainty Scores T2	28	0.640**	0.664**	0.757**	-0.434*	-0.637**	0.576**	__

**. Correlation is significant at the 0.01 level (2-tailed)

*. Correlation is significant at the 0.05 level (2-tailed)

**Table 9:** Content analysis of qualitative questions on interest and the parent’s favourite part

Orientation	Focus	First interest question (%)	Favourite part question (%)	Total (%)
**Social**	Parent Needs	5 (22.7%)	3 (14.3%)	8 (18.6%)
	Child Needs	5 (22.7%)	4 (19.0%)	9 (20.9%)
	Both	12 (54.5%)	14 (66.7%)	26 (60.5%)
	Neither	0 (0%)	0 (0%)	0 (0%)
	Total	22	21	43
**Not Social**	Parent Needs	1(4.3%)	1(8.3%)	2 (5.7%)
	Child Needs	10 (43.4%)	4 (33.3%)	14 (40.0%)
	Both	4 (17.3%)	3 (25.0%)	7 (20.0%)
	Neither	8 (34.7%)	4 (33.3%)	12 (34.3%)
	Total	23	12	35

Note.There were 45 responses for the first interest question and 33 responses for the favourite part questions. All questions were coded for both social orientation and needs

## Discussion

This is the first study to assess parental mental health outcomes following a SP creative play intervention, and thus offers a novel contribution and original results, which have broad applicability and international relevance. The participants and their children took part in a 5-week creative play on prescription programme, designed to offer opportunities to build social connections and social support, improve parenting confidence and mental health, and develop RF. This was in line with the social cure model of SP [56]. The findings can be summarised thus: The participants experienced a statistically significant reduction in postnatal depression scores following the intervention; secondly, birth trauma scores predicted T2 depression scores as well as RF uncertainty scores (but not RF certainty scores). The results partially support the hypotheses and intervention efficacy where birth trauma did indeed have relationships with depression and RF, and depression symptomology scores lowered, however there was no change in RF scores Table 10.

**Table 10:** Content analysis of qualitative questions on differences and parent help

	Group difference question	Parent help question	Total
Trust	5	7	12
Calm	8	6	14
Practical Parenting Knowledge	3	6	9
Music & Play	7	3	10
Intimate and personalised	15	4	19

Note: A total of 30 participants answered the group difference questions and 21 participants answered the parent help questions. Some answers did not fit into the coding scheme

The postnatal depression symptomology scores did indeed decrease through the 5-week intervention. This was regardless of whether the parent had attended the SP creative play intervention for the first time or had attended with another cohort previously. These results are in line with other parenting interventions which have been found to be associated with decreases in postnatal depression symptomology (e.g. Tsivos et al., 2015). Past interventions include those designed to focus on parent knowledge, confidence, skills, psychoeducation and even infant massage [69–71]. In future, it would be important to focus on the constellation of specific elements that might make the interventions successful as the SP feature combined with the ethos and creative play prompts, infant development and professionally curated music elements are unique to this intervention. The concepts of creative play may also be more normalized and salient for new parents, thus improving uptake.

Although depression scores fell significantly after the intervention, they were still able to be predicted by the baseline birth trauma PTSD symptom report. These results are congruent with other reports of comorbid rates of postnatal depression [87–89]. Future iterations of this intervention should work on being trauma informed and consider that even if parents are accessing the SP creative play, that they can still feel vulnerable or disempowered when involved in the intervention [90]. Goodman and colleagues [91] found that when considering trauma informed practice there are six dimensions in assessing survivors’ experiences and these were agency, information, connection, strengths, inclusivity and parenting, specifically support for parenting. Future SP creative play interventions could adopt a trauma informed approach where providers are knowledgeable that parents engaging in the SP intervention might be birth trauma survivors and the recognition along with parental support is important for recovery.

Birth trauma PTSD scores also predicted RF uncertainty scores after the intervention. Past research has found that uncertain RF is related to trauma while certain RF is not [62]. RF certainty and uncertainty scores were not significantly changed through the SP creative play intervention which supports the meta-analysis performed by Barlow and colleagues [6] which found only trends for moderate improvement in parental reflective function while postnatal depression showed large impacts. The RF certainty means did increase T1 to T2, however not significantly. This could have been because of the low participant numbers or short duration of the programme. In future it would be important to recruit for a larger scale study.

Qualitative analysis of social opportunities revealed that parents originally may not have entered the group for the social aspects, however after being involved, their favourite parts were social. The non-social responses around why parents entered the group and what they enjoyed focused more on their child’s needs (also confirmed through chi-square analysis) while the more social responses focus were parent or the needs of both parent and child. The themes of trust, calm and practical parenting knowledge were all present when mentioning the programme, along with themes of musicality, play and intimacy, personalization and a child-led nature. The qualitative component of the study informed researchers about the value of the social opportunities parents received through the SP intervention. Many may not have realised that this was a factor when signing up, and instead were focusing on more practical reasons to attend, like the ability to bring both of their children, or it being recommended and at the correct time. After attending parents tended to assign the social factors as their favourite parts of the SP creative play intervention. The social capital gained through SP creative play was a key focus as this is a goal of SP programmes in general [92, 93]. There was a statistical association found between parents citing non-social opportunities tending to talk about their children’s needs over their own. These needs were in reference to learning songs, rhythm, creative playing or learning to speak. Those that did report social aspects explained that they valued making friends, socialising and seeing the children play with others.

The qualitative questions not only revealed trends in the reports of social capital, but also showed that parents are coming back to the SP creative play because of the trust that they have in the people and organization. They are also finding the intervention helpful from a parenting information perspective. This result supports previous evidence that the trust and calm of SP creative play can increase practical parenting knowledge uptake [47]. The other two themes that were evident in the content analysis were that of music and the enjoyment both parent and child gleaned from singing and the personalised, small, child-centered feel of the programme on a whole. The personalised one-to-one nature could have also been a factor in the knowledge exchange through parents and the arts professional leading the group. It has been found that parents enjoy groups where connections are made [94]. Furthermore, the music aspect has been found to benefit children and could also impact attachment [95].

The results of this investigatory study should be considered in light of some limitations. The first is that although the postnatal depression symptomology significantly decreased from T1 to T2, there is no evidence for the SP programme to be causal in this change. There are many variables that could be contributing to depression symptomology outside of the programme. Furthermore inside the programme there are also factors to consider. Future research should examine what elements of the SP creative play intervention in particular may be influencing the changes in postnatal depression symptomology report. Among the potential contributing factors, the creative play itself, the social opportunities, the musical aspect of the programme or the personalization could influence the scores. It would be useful to perform some modest trials around SP creative play using a play group as a control and to examine other factors in depth by using validated scales of social support, parenting confidence or identity.

Another limitation is that there were only 28 participants that responded to both questionnaires. This was partly because a number of participants did not remember or add in their unique pseudonym to link the two timepoints. There were also some participants that did not attend either T1 or T2. As we were interested in the effect of the creative play intervention on parental mental health on individual bases (rather than comparing the creative play intervention to another type of intervention), the repeated measures ANOVA effectively controls for individual differences, reduces error variance, controls for type 1 error and excellent statistical power was achieved (0.97), However, we acknowledge the low participant number and recognise the potential for low statistical power and issues for generalisability in the regression analyses, which may explain why RF certainty scores did not change significantly although means rose. As there were no systematic differences between those who completed one, both or only one assessment point, we believe there is little concern for response bias arising from missing data. Finally, other potential confounding factors (e.g. family support systems, access to mental health services, and parental stress levels) that were not accounted for could also be important in explaining the pattern of results that were found. One way forward for this programme would be to perform a realist evaluation to determine how SP creative play works, for whom and under what conditions [96].

The findings from this pilot study suggest SP creative play holds positive outcomes for parents/caregivers of young children. The next step would be to conduct a two-group experimental design, incorporating a control group to assess differences in outcomes between groups. Including potential mediators like parental creativity and playfulness. Future research may also consider using a measure of parental RF, for specific mentalisation processes within parent/caregiver-child relationships. One consideration for future studies is that both RF and social support could also be mediators rather than outcome variables. Parents could bring these into the programme thus the extent to which these are further developed during the session could impact outcomes like postnatal depression. Presently, there is a baseline RF score, but in future, having a social support scale would improve the study. This is in line with the social cure framework as group belonging and social relationships are drivers of health outcomes.

## Conclusion

The transition to parenthood is a period of significant change, including changes to identity, social roles, and daily routines [97]. This time period has been argued to be a sensitive period in health behaviour change which could include healthy diet, physical activity and mental health [98]. By prescribing creative play, health stakeholders can promote healthy wellbeing and connection [99]. Having creative play as an option is an opportunity to help tap into this period and not only shape health and wellbeing behaviours, but also shape and change parent identity to that of a creative, playful parent that knows how to connect to their child through the play medium. Considering wait times for NHS services, this programme could be a part of a suite of “waiting well” interventions for new parents.

On a whole, this pilot study indicates that SP creative play provides parents with social capital that they value. The parents involved in the programme are showing improved postnatal depression symptomology even if they are showing birth related PTSD and this is regardless of whether they attend 1 or more 5-week interventions. The continued significant changes in the depression scores indicate that longer 12-week sessions might be more helpful to parents. Furthermore, RF scores did not show significant changes, but might with a longer session and more emphasis on perspective taking by the practitioner. This type of an intervention is promising for new parents who may be struggling with mental health difficulties.

## Electronic supplementary material

Below is the link to the electronic supplementary material.

Supplementary Material 1

## Data Availability

Data is provided within the manuscript or supplementary information files.

## Abbreviations

SP: Social Prescribing/prescription
T1: Baseline
T2: Post intervention
RF: Reflective function
EPDS: Edinburgh Postnatal Depression Survey
PTSD: Post traumatic stress disorder
