# Supplementary material for: A socially prescribed creative play intervention for new parents: investigating post traumatic stress around birth and changes in postnatal depression and reflective function
Source: BMC Psychol. 2025 Mar 23;13:291. doi: 10.1186/s40359-025-02578-3 (PMC11931792; doi:10.1186/s40359-025-02578-3)
Supplement: Supplementary file 1 — Supplementary Material 1 [file 40359_2025_2578_MOESM1_ESM.docx]

**Appendices**

**Appendix 1**

**Table 1**

*Mean differences in those who had previously attended the creative play intervention and those who were new participants*

|  | Previous intervention  (n = 17)  Mean (SD) | First intervention  (n = 28)  Mean (SD) | *t* statistic (df) | *p* value |
| --- | --- | --- | --- | --- |
| Parent age | 31.88 (6.17) | 30.18 (6.42) | -.88 | .39 |
| Child age (months) | 16.06 (9.70) | 12.38 (8.08) | -1.33 | .19 |
| Sum EPD T1 | 9.94 (5.39) | 11.48 (4.99) | .98 | .33 |
| 13-22 symptoms T1 | 8.53 (9.06) | 8.88 (7.75) | .13 | .89 |
| Sum RFQC T1 | .87 (.84) | 1.09 (.65) | 1.03 | .31 |
| Sum RFQU T1 | .64 (.72) | .56 (.48) | -.45 | .66 |
| Sum RFQC T2 | 1.23 (.67) | .92(.96) | -.97 | .34 |
| Sum RFQU T2 | .33 (.61) | .53 (.50) | .88 | .39 |
| Sum EPD T2 | 7.93 (7.44) | 10.09 (7.12) | .74 | .46 |

**Table 2**

*Sample characteristics with chi-square analyses to examine previous intervention attendance differences*

|  | Total | | Previous intervention | | First intervention | | *X^2^* |
| --- | --- | --- | --- | --- | --- | --- | --- |
| Variables | *n* | % | *n* | % | *n* | % |  |
| Child gender |  |  |  |  |  |  |  |
| Male | 27 | 58.7 | 9 | 52.9 | 18 | 62.1 | .37 |
| Female | 19 | 41.3 | 8 | 47.1 | 11 | 37.9 |  |
| Income |  |  |  |  |  |  |  |
| < 29k | 16 | 40 | 6 | 35.3 | 10 | 43.5 | .27 |
| > 30k | 24 | 60 | 11 | 64.7 | 13 | 56.5 |  |
| Ethnicity |  |  |  |  |  |  |  |
| Majority | 39 | 86.7 | 13 | 76.5 | 26 | 92.9 | .13a |
| Minority | 6 | 13.3 | 4 | 23.5 | 2 | 7.1 |  |

*Note.* a= Fisher's exact test

**Appendix 2**

*Correlation Table for Parent and Child Age investigating Relationships with Birth Trauma, Postnatal Depression and Reflective Function*

| Variable | *n* | 1 | 2 | 3 | 4 | 5 | 6 | 7 | 8 | 9 |
| --- | --- | --- | --- | --- | --- | --- | --- | --- | --- | --- |
| 1. Parent   Age | 48 | __ |  |  |  |  |  |  |  |  |
| 2. Youngest Child Age | 44 | .403** | __ |  |  |  |  |  |  |  |
| 3. Birth Trauma Scale T1 | 43 | -.165 | -.166 | __ |  |  |  |  |  |  |
| 4. RFQ Certainty Scores T1 | 48 | .064 | -.224 | -.420** | __ |  |  |  |  |  |
| 5. RFQ Certainty  ScoresT2 | 28 | .015 | -.072 | -.431* | .648** | __ |  |  |  |  |
| 6. RFQ Uncertainty Scores T1 | 48 | -.061 | .156 | .478** | -.680** | -.576** | __ |  |  |  |
| 7. RFQ Uncertainty Scores T2 | 28 | -.140 | -.084 | .640** | -.434* | -.664** | .576** | __ |  |  |
| 8. EPDS  T1 | 48 | -.230 | -.080 | .569** | -.467** | -.715 | .645** |  | __ |  |
| 9. EPDS  T2 | 27 | -.241 | -.054 | .650**. | -.281 | -.597** | .486* | .749** | .781** | __ |
